# A distinctive new subspecies of *Scytalopus griseicollis* (Aves, Passeriformes, Rhinocryptidae) from the northern Eastern Cordillera of Colombia and Venezuela

**DOI:** 10.3897/zookeys.506.9553

**Published:** 2015-06-02

**Authors:** Jorge Enrique Avendaño, Thomas M. Donegan

**Affiliations:** 1 Programa de Biología y Museo de Historia Natural, Universidad de los Llanos, Sede Barcelona, km 12 vía Puerto López, Villavicencio, COLOMBIA; 2 ProAves Foundation, Southmead, The Vale, London N14 6HN, UK

**Keywords:** Andes, endemism, geographic variation, inter-Andean valley, páramo, Tamá

## Abstract

We describe a new subspecies of Pale-bellied Tapaculo *Scytalopus griseicollis* from the northern Eastern Cordillera of Colombia and Venezuela. This form differs diagnosably in plumage from described subspecies *Scytalopus griseicollis griseicollis* and *Scytalopus griseicollis gilesi* and from the latter in tail length. It is also differentiated non-diagnosably in voice from both these populations. Ecological niche modelling analysis suggests that the new subspecies is restricted to the Andean montane forest and páramo north of both the arid Chicamocha valley and the Sierra Nevada del Cocuy.

## Introduction

*Scytalopus* Tapaculos are a genus of small and dull suboscine passerines which inhabit the undergrowth of humid forests and tree-line habitats of Neotropical mountains from Costa Rica to Argentina (Krabbe and Schulenberg 2003). *Scytalopus* taxonomy has been challenging due to the lack of plumage differences among species, scarcity of specimens and historically poor knowledge of their distributions and vocalisations (Krabbe and Schulenberg 1997, 2003, Cuervo et al. 2005). Recent studies have resulted in new taxon descriptions and reclassifications for at least 30 species (principally in Fjeldså and Krabbe 1990, Krabbe and Schulenberg 2003 and in many other publications, see Remsen et al. 2015). Six of these new *Scytalopus* taxa have been described with a distribution partially or exclusively in Colombia since the late 1990s (Krabbe and Schulenberg 1997, Cuervo et al. 2005, Krabbe et al. 2005, Donegan and Avendaño 2008, Donegan et al. 2013, Avendaño et al. 2015) and some diagnosable populations in the Colombian Andes remain undescribed (e.g. Donegan and Avendaño 2008, López-O et al. 2013, McMullan and Donegan 2014).

The Pale-bellied Tapaculo *Scytalopus griseicollis* inhabits subpáramo and páramo habitats of the Eastern Cordillera (Eastern Andes) of Colombia and Venezuela (Hilty and Brown 1986, Donegan and Avendaño 2008). We recently reviewed the taxonomy of the group and related taxa (Donegan and Avendaño 2008), clarified the status and affinities of the type specimens of various names from the region, addressed the previously controversial status of *Scytalopus infasciatus* as a synonym of nominate *Scytalopus griseicollis* and described *Scytalopus griseicollis gilesi* from the Serranía de los Yariguíes of the Eastern Cordillera of Colombia. Two named subspecies and, in some cases, an additional undescribed subspecies have been recognised by subsequent authors who have considered the group’s taxonomy (Salaman et al. 2008, 2009, 2010, McMullan and Donegan 2014, Dickinson and Christidis 2014). We identified a further population as “*Scytalopus griseicollis* *subsp.*” which we considered to possess a “notably browner back in adult plumage than any of the other populations” (at Fig. 9, p. 39). This population was mapped as present in the northern section of the Eastern Cordillera from Santander and Norte de Santander departments in Colombia to Apure and Táchira states in Venezuela. Vocal, plumage and biometric data relating to this population were presented but it was not described.

## Methods

A total of 88 specimens of *Scytalopus griseicollis* from eight museum collections, reviewed in Donegan and Avendaño (2008), plus another 23 recently collected and 12 from the Smithsonian National Museum of Natural History (USNM) were inspected directly or using photographs in order to investigate geographical variation in plumage and biometrics of *Scytalopus griseicollis* (Suppl. material 1). Specimens of the northern population were collected by JEA at seven localities in Santander department as part of various different projects (see Acknowledgments). In all localities, specimens were collected using mist nets and air shotgun cal. 4.5. We made color descriptions in the description of the holotype and variation in the type series using Smithe’s (1975, 1981) colour nomenclature. Biometric and vocal data for several characters and each of the three same populations are based in Donegan and Avendaño (2008) and followed various statistical tests of diagnosability as set out in that reference.

With the aim of obtaining a more detailed assessment of the potential distribution of the new subspecies, we conducted an ecological niche modelling analysis in the program Maxent version 3.3 (Phillips et al. 2006), using 19 climate variables available in the WORLDCLIM ver. 1.4 database (Hijmans et al. 2005) and 13 remote-sensing variables related to vegetation and three related to topography (Buermann et al. 2008). The analysis was conducted for *Scytalopus griseicollis* as a whole, including 59 locality points, which were gathered from museum specimens, sound recordings, and reliable field observations that counted with geographic coordinates (to seconds) and elevation data. Geographic coordinates of all known localities are summarized in Suppl. material 2.

## Results

Previous biometric and vocal analyses (Donegan and Avendaño 2008), combined with recent specimens and new analyses of plumage and ecological niche modelling, reinforce the conclusion that the population of *Scytalopus griseicollis* in the northern Eastern Cordillera represents a previously undescribed subspecies, which we propose be named:

### Scytalopus griseicollis morenoi

Taxon classificationAnimaliaPasseriformesRhinocryptidae

Avendaño & Donegan ssp. n.

http://zoobank.org/70DE583A-AC75-466D-B3FB-84B1046BBBBB

#### Holotype.

**Figure 1.**
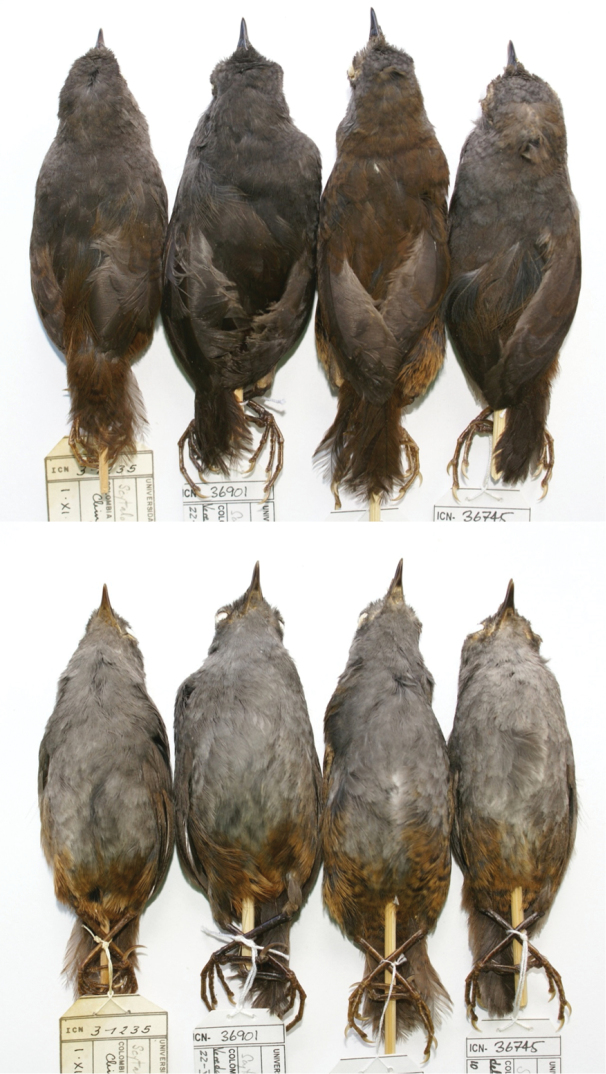
Dorsal and ventral views of three subspecies of *Scytalopus griseicollis* found in the Eastern Cordillera of Colombia and Venezuela and *Scytalopus perijanus* from the Serranía de Perijá. From left to right. *Scytalopus griseicollis griseicollis* (ICN 31235); *Scytalopus griseicollis gilesi* (ICN 36901); *Scytalopus griseicollis morenoi* (holotype); and *Scytalopus perijanus* (ICN 36745). Note the distinctive browner back and nape of the new subspecies.

An adult male study skin specimen (Figs 1 and 3), no. 37538 of the ornithological collection of the Instituto de Ciencias Naturales (ICN), Universidad Nacional de Colombia, Bogotá. Tissue samples (pectoral muscle) are deposited at the Banco de Tejidos of Universidad de los Andes (ANDES-BT 1567), Bogotá. The specimen and tissue samples relate to the same individual organism which was collected and prepared on 2 August 2009 by J. E. Avendaño (original field no. JEA 811) in secondary growth / forest ecotone at La Pica, finca La Rinconada, vereda Potrero de Rodríguez, municipality of Molagavita, Santander department, Colombia (06°43'N; 72°47'W; 2880 m).

#### Diagnosis.

**Figure 2.**
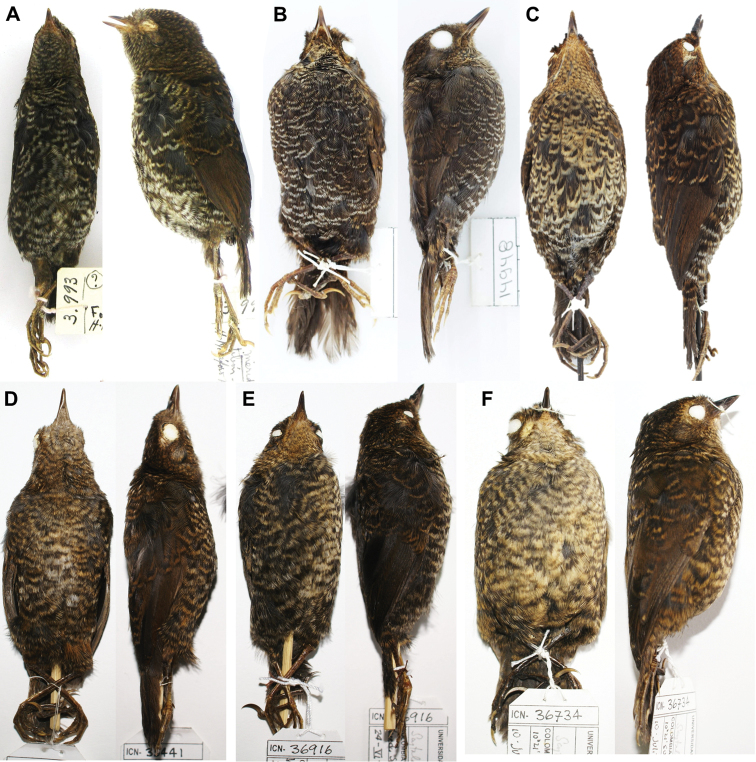
Ventral and lateral views of fledglings of three subspecies of *Scytalopus griseicollis* and *Scytalopus perijanus* found in Colombia and Venezuela. **A** *Scytalopus griseicollis morenoi* (MLS 3993) **B** *Scytalopus griseicollis morenoi* (IAvH-A 14948); **C** *Scytalopus griseicollis griseicollis* (IAvH-A 13935) **D** *Scytalopus griseicollis griseicollis* (ICN 35441) **E** *Scytalopus griseicollis gilesi* (ICN 36916) **F** *Scytalopus perijanus* (ICN 36734). Note the darker plumage and ventral white scalloping in *Scytalopus griseicollis morenoi*.

*Scytalopus griseicollis morenoi* exhibits all the characteristics of the genus *Scytalopus* (Ridgway 1911, Krabbe and Schulenberg 1997, Cuervo et al. 2005). It appears to be most closely related to *Scytalopus griseicollis* on account of its rather grey plumage, orange-rufous vent (Fig. 1) and similar vocalisations (Figs 4–5). *Scytalopus griseicollis morenoi* is distinguishable from *Scytalopus perijanus* from the Serranía de Perijá by its entirely brown nape and back (Fig. 1) and distinct vocalisations (Avendaño et al. 2015). It is diagnosable from *Scytalopus griseicollis griseicollis* of the Altiplano Cundiboyacense and *Scytalopus griseicollis gilesi* of the Serranía de los Yariguíes by its brown (not grey) mantle, tail, wing coverts and nape (Fig. 1). Juveniles of the new subspecies differ mainly from the nominate and *Scytalopus griseicollis gilesi* in having darker base plumage ventrally (which is scalloped white) whereas they are dorsally darker than the nominate, like in *Scytalopus griseicollis gilesi* (Fig. 2). These characters also distinguish juveniles of the new subspecies from those of *Scytalopus perijanus*, which are more yellowish ventrally. It also has a shorter tail than *Scytalopus griseicollis gilesi* (see Appendices 2 and 4 in Donegan and Avendaño (2008)). It has an on average higher pitched scold than *Scytalopus griseicollis griseicollis* but this is not diagnostic. Compared to *Scytalopus griseicollis gilesi*, the new subspecies has a faster and higher frequency song and higher frequency scold (in the latter case, with no overlap) (Figs 4–5; and Appendices 3–4 in Donegan and Avendaño (2008)).

**Figure 3.**
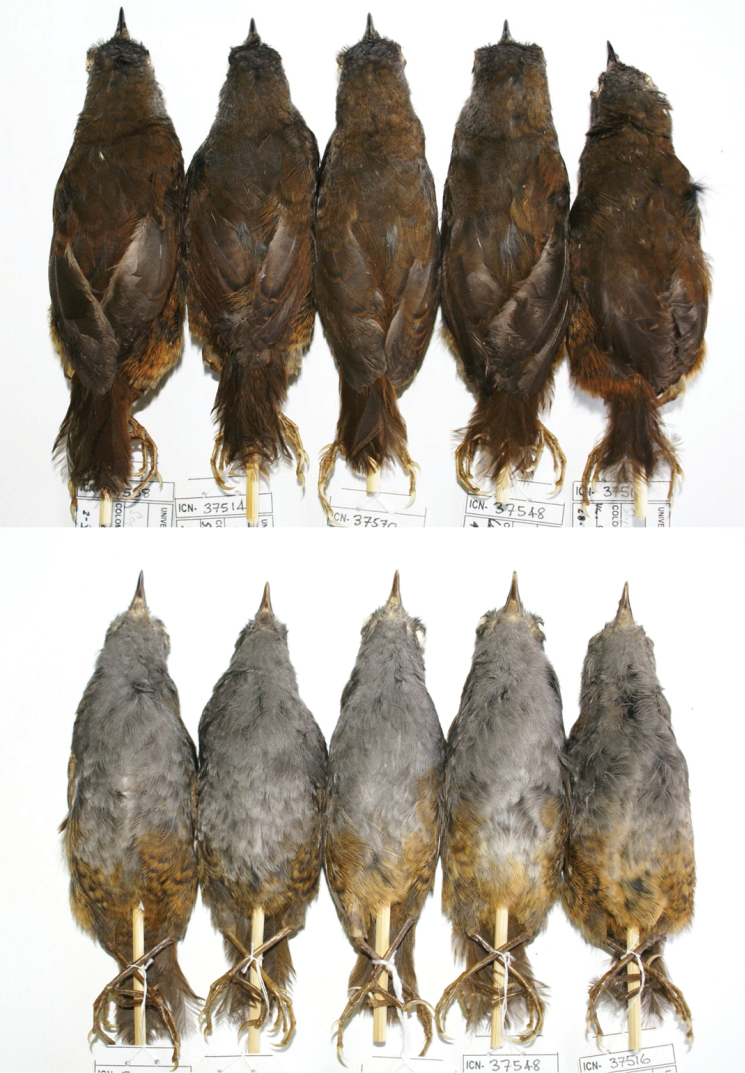
Selected specimens of the type series of *Scytalopus griseicollis morenoi*. From left to right: holotype (ICN 37538), male paratype (ICN 37514), male paratype (ICN 37570), male paratype (ICN 37548), and female paratype (ICN 37516). Note the slight individual variation in the color of the underparts and upperparts.

**Figure 4.**
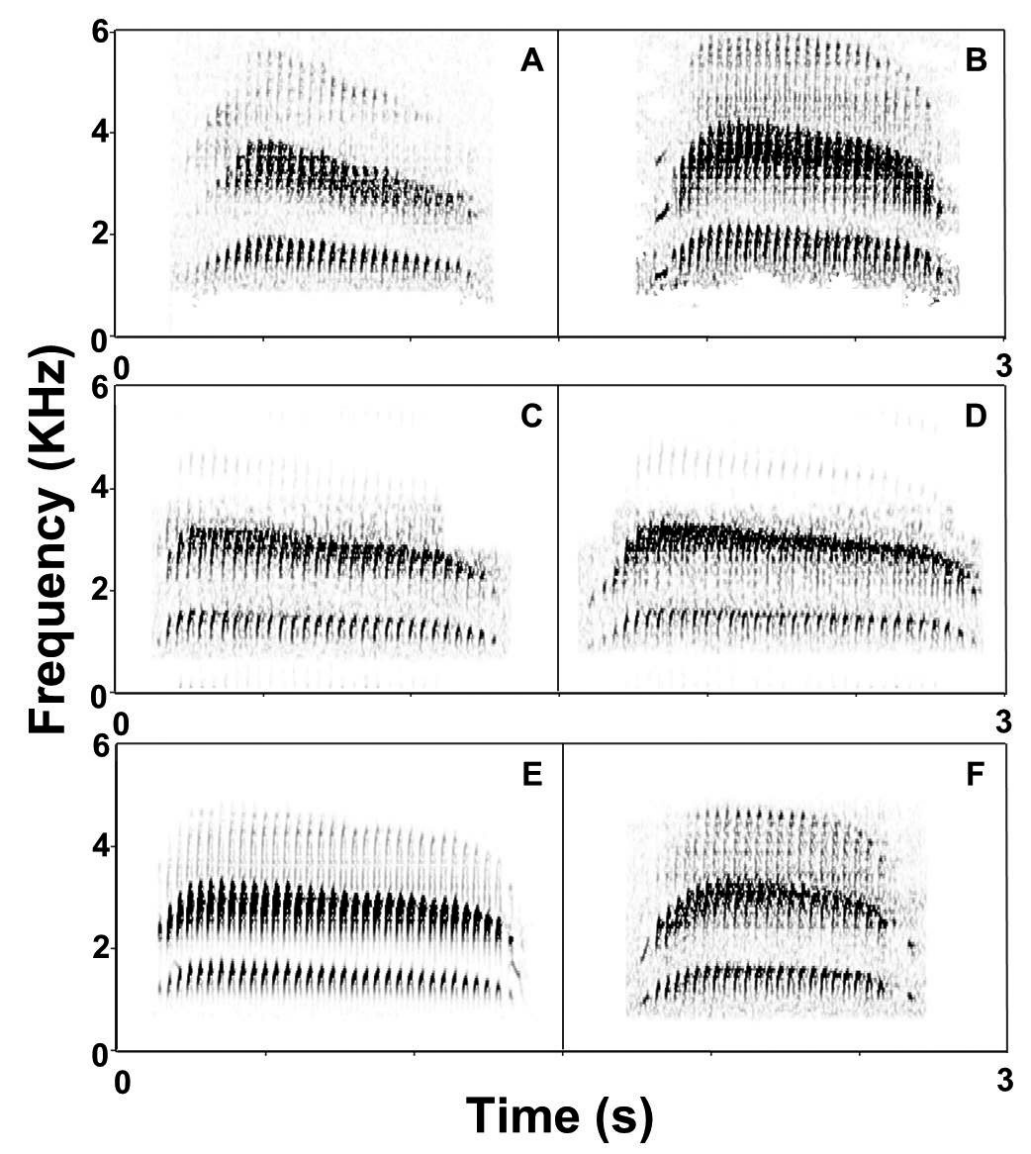
Spectograms of scolds of *Scytalopus griseicollis* subspecies found in Colombia and Venezuela. *Scytalopus griseicollis morenoi*: **A** Oirá River, border with Colombia, Apure state, Venezuela (XC6079, C. Parrish) **B** Páramo de Santurbán, Vetas, Santander department, Colombia (XC117002, O. Cortés). *Scytalopus griseicollis gilesi* **C** vereda Alto Cantagallos, San Vicente de Chucurí, Santander department, Colombia (XC18457, T. M. Donegan) **D** Lepipuerto, El Carmen de Chucurí/Simacota, Santander department, Colombia (XC18477, T. M. Donegan). *Scytalopus griseicollis griseicollis* **E** Chingaza NP, Cundinamarca department, Colombia (XC79989, A. Spencer) **F** Iguaque, Boyacá department, Colombia (XC119700, D. Edwards). Spectrograms were made in Syrinx v2.6h (Burt 2006) applying the same parameters except for adjusting brightness to improve note resolution.

**Figure 5.**
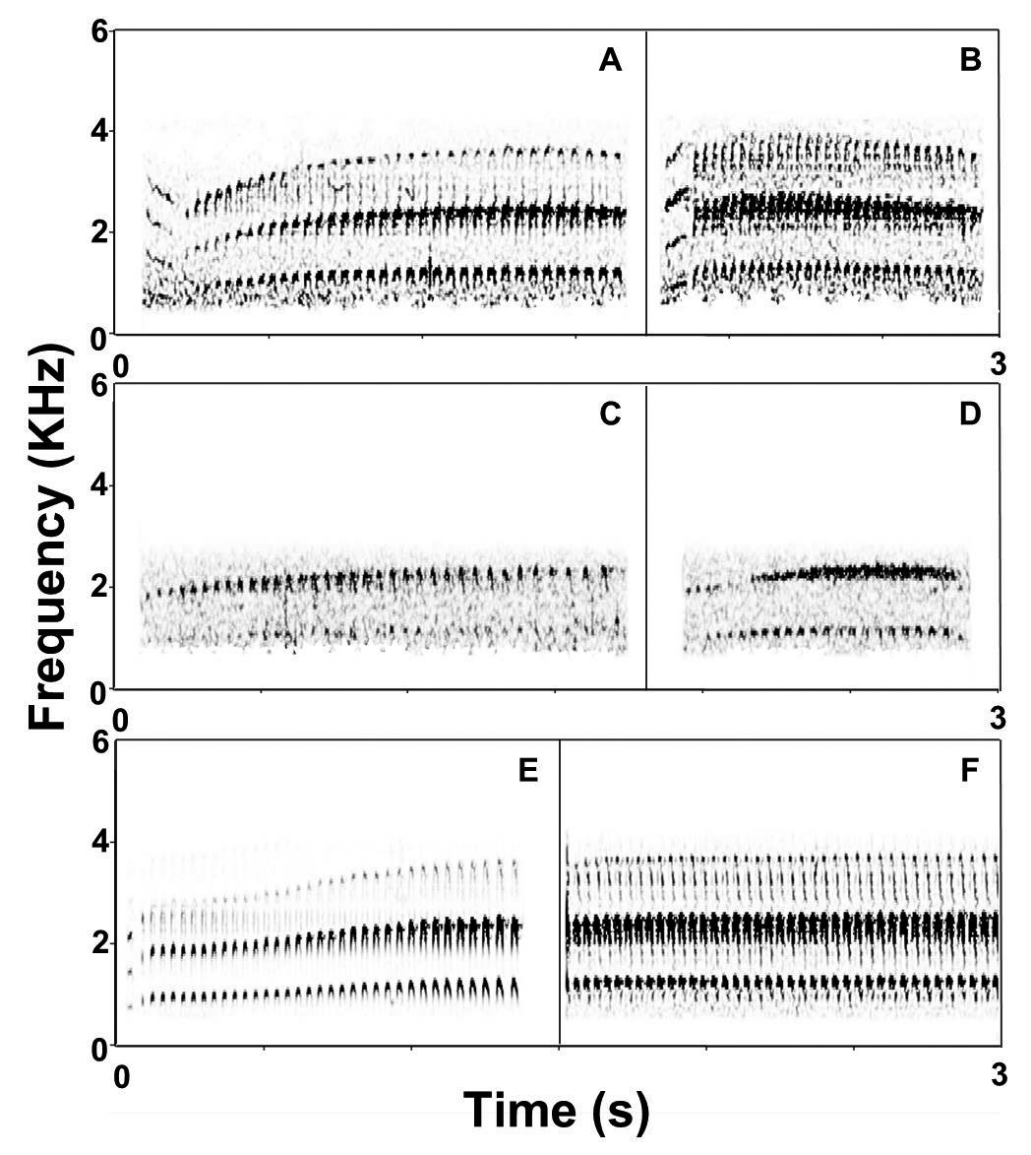
Spectograms of reeling songs of *Scytalopus griseicollis* subspecies found in Colombia and Venezuela. *Scytalopus griseicollis morenoi*: **A** Las Picotas, vereda Angosturas, Vetas, Santander department, Colombia (XC86713, J. E. Avendaño) **B** Oirá River, border with Colombia, Apure state, Venezuela (XC16658, C. Parrish). *Scytalopus griseicollis gilesi* **C** Filo Pamplona, vereda La Aurora, Galán, Santander department, Colombia (XC18454, T. M. Donegan) **D** Lepipuerto, El Carmen de Chucurí/Simacota, dpto. Santander, Colombia (XC18472, T. M. Donegan). *Scytalopus griseicollis griseicollis* **E** Chingaza NP, dpto. Cundinamarca, Colombia (XC102520, F. Schmitt) **F** (first part of song) Reserva de Aves para *Amazilia castaneiventris* y *Macroagelaius subalaris*, Soatá, dpto. Boyacá, Colombia (XC94523, O. Cortés).

#### Description of the holotype.

Lores, forehead, crown, auriculars and neck sides Dark Neutral Gray 83; nape, scapulars, mantle, rump, tail and upper-tail coverts between Verona Brown 223B and Amber 36, the latter barred with Sepia 219. Underparts Medium Neutral Gray 84, becoming slightly lighter (Light Neutral Gray 85) on the center of belly; flanks, lower belly, thighs and under-tail coverts between Buff 24 and Tawny 38, the latter barred with Sepia 29. Wing coverts Dark Neutral Gray 83 fading to Dark Brownish Olive 129 and tipped with Verona Brown 223B; remiges and tertials Dark Grayish Brown 20 with external margin Cinnamon-Rufous 40, and the latter tipped Tawny 38, with dark (Sepia 119) subterminal bar. Light molt in mantle, throat, breast and abdomen. 10 rectrices. Measurements (in mm): wing flat 56.0, tail 41.1, tarsus 20.5, total culmen 13.2, exposed culmen 9.9. Mass 17.0 g. During preparation and dissection, the following features were noted, none of which is evident from the holotype itself: some subcutaneous fat in furcula and neck; testes rather enlarged (left testis: 5.3 × 2.5 mm; right testis 4.8 × 3.0 mm); stomach contained insect remains. Soft parts in life (not coded for colours in the field): bill dark (‘horn’), lighter on the base of the lower mandible; iris dark brown; tarsus and feet light brown, claws whitish, hallux blackish, soles pale yellow.

#### Paratypes.

The type series includes the following specimens in museums which we have been able to compare directly with fresh specimens collected as part of this study. The specimens showed in Figures 2–3 are denoted with an asterisk. (1) Adult male (ICN 37548*) collected at the type locality on 4 August 2009; (2) adult male (ICN 37514*) collected at 2700 m elevation above finca La Paterna, vereda San Isidro, corregimiento of Pangote, municipality of San Andrés, Santander department, on 28 July 2009; (3) adult male (ICN 37570*) collected at 2800 m elevation at finca El Tablón, vereda Santa Cruz, municipality of San Andrés, Santander department, on 14 September 2009; (4) adult male (ICN 36121) collected at 2950 m elevation at El Gritadero, vereda El Monsalve, municipality of Suratá, Santander department, on 19 August 2006; (5) adult male (ICN 36416) collected at 3100 m elevation at finca Ramírez, vereda Parra Juan Rodríguez, municipality of Piedecuesta, Santander department, on 11 July 2007; (6) adult female (ICN 37516*) collected at 2725 m elevation at La Corcova, vereda San Isidro, municipality of San Andrés, Santander department, on 28 July 2009; (7) adult female (ICN 37522) collected at 2950 m elevation below Pozo El Indio, La Pica, finca La Rinconada, Vereda Potrero de Rodríguez, municipality of Molagavita, Santander department, on 30 July 2009; (8) fledgling male (IAvH-A 14948*) collected at 2800 m elevation by S. Sierra at Alto El Pesebre, Sector Orocué, Tamá NP, municipality of Herrán, Norte de Santander department, on 18 September 2008; (9) unsexed fledgling (MLS 3993*) collected by Hno. Nicéforo María at Fontibón, municipality of Pamplona, Norte de Santander, on 30 April 1941. Specimens 1 to 7 were collected and prepared by J. E. Avendaño under original field numbers JEA-821, 787, 916, 323, 499, 789 and 795, respectively. See further Suppl. material 1.

#### Etymology.

The new subspecies name honours the late Nelson Moreno Rodríguez, co-founder and curator of the Museo de Historia Natural of the Universidad Industrial de Santander. He was a mentor and friend of the first author and an enthusiastic naturalist. This name also recognizes his contributions to ornithology, natural history and education in the department of Santander. The name is formed from a fictional masculine Latin noun “morenous”, in the genitive singular. The name is non-variable.

#### Remarks.

**Variation in the type series.** Plumage variation in the type series is slight and mainly concentrated in the colour tone of the nape, back and underparts (Fig. 3). Males ICN 37548 and 37570 have paler underparts (Pale Neutral Gray 86), the former with a whitish tinge in the center of the belly; both specimens have flanks, lower belly, thighs and under-tail coverts more tawny than the holotype. Male ICN 37514 is slightly paler on the belly. Some males (e.g. ICN 37514, 37548 and 37570) are duller (less Amber 36) than the holotype and show some grey (Dark Neutral Gray 83) feathers in the nape and mantle. Both females at ICN are ventrally similar to the holotype, but ICN 37516 has a tinge of Pale Pinkish Buff 121D in the belly. Nape and back coloration differs from the holotype as in males ICN 37514, 37548 and 37570. Juvenile specimens, such as MLS 3993 and IAVH-A 14948, have very dark brown base coloration with narrow, scallopped whitish markings on the trailing edges of all head, underparts, dorsal and wing covert feathers (Fig. 2). Details on vocal and biometric variation are presented in the appendices to Donegan and Avendaño (2008).

#### Additional specimens examined.

We examined 42 additional specimens we identified as *Scytalopus griseicollis morenoi* (Suppl. material 1). All these specimens exhibit variation within the range described above for the type series.

#### Distribution.

**Figure 6.**
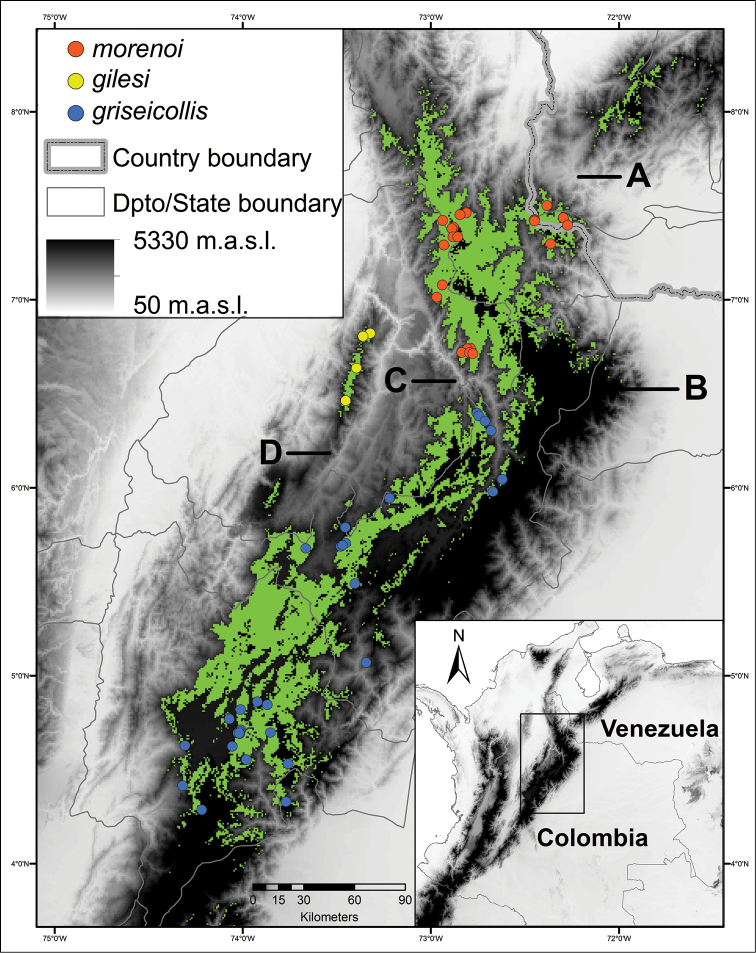
Potential distribution (in green, defined as ≥0.44 presence probability calculated in MAXENT) for three subspecies of *Scytalopus griseicollis* in the Eastern Cordillera of Colombia and Venezuela. Note the restricted potential range of *Scytalopus griseicollis morenoi* to the northern section of the Eastern Cordillera. Bold letters correspond to some potential barriers or geographic locations mentioned in the text: **A** Táchira depression **B** Sierra Nevada del Cocuy **C** Chicamocha River canyon; and **D** Horta-Opón Rivers depression. Locality records by subspecies are depicted by colored circles.

*Scytalopus griseicollis morenoi* is endemic to the northern Eastern Cordillera in Colombia and Venezuela, ranging from La Palmita in Norte de Santander, south to Molagavita in Santander, and covering an altitudinal range between 2000 m and 3900 m (Suppl. materials 1–2; Participantes de la Alianza Biomap 2014). Our niche model suggests that *Scytalopus griseicollis morenoi* is present on both slopes of the northern section of the Eastern Cordillera, largely in more humid montane slopes and subpáramo to páramo habitats (Fig. 6). Range limits in *Scytalopus griseicollis*’ subspecies seem to correspond to several geographic barriers and changes in environmental conditions across the northern Eastern Cordillera (Graham et al. 2010). Northern distributional limit of *Scytalopus griseicollis morenoi* seems to concur with the Ocaña (Serranía de los Motilones) depression (*c.*1200 m). Its distribution to the north-east is restricted by the Táchira depression, despite our niche model predicting some suitable habitat in the southern Mérida Cordillera (Fig. 6). The vocally distinctive and ecologically less specialised *Scytalopus meridanus* replaces the species in the Venezuelan Andes (Donegan and Avendaño 2008, Hilty 2003). These barriers prevent contact of a high elevation specialist with poor dispersal abilities, such as this, with *Scytalopus perijanus* of the Serranía de Perijá and *Scytalopus meridanus* of the Mérida Cordillera, respectively. On the east slope of the Eastern Cordillera, the distribution of *Scytalopus griseicollis morenoi* seems to be restricted to the Tamá-Sierra Nevada del Cocuy foothills. This region appears to constitute the northern or southern distribution limit of many montane species and subspecies on the east slope of the Eastern Cordillera (Hilty and Brown 1986, Restall et al. 2006).

To the south, *Scytalopus griseicollis morenoi*’s potential habitat becomes reduced and discontinuous, possibly related with an environmental break at the head of the arid Chicamocha valley in the Santander-Boyacá departments boundary, which precludes any potential contact with the nominate form of the Altiplano Cundiboyacense. The headwaters of the arid Chicamocha valley represent the northern or southern distributional limit of several montante and páramo species ranging along the west slope of the Cordillera or represent internal breaks of widely distributed species (Hilty and Brown 1986, Graham et al. 2010). Likewise, the new subspecies is isolated from *Scytalopus griseicollis gilesi* by a depression at the headwaters of the rivers Horta and Opón, which connects the Serranía de Yariguíes with the rest of the Eastern cordillera (c. 1450 m).

#### Ecology.

A typical dweller of the understory of elfin forest, páramo and rarely montane and oak forest, although it also can be found at forest borders and bushes. Individuals, possibly young birds were occasionally observed crossing small pastures and trails between patches of more appropriate habitat. *Scytalopus griseicollis morenoi* has been recorded as fairly common to common at several localities in Santander with 2-4 individuals recorded in 1 ha study sites (J.E.A. pers. obs.). Breeding and reproduction may take place during the second half of the year. Fledglings have been collected at Tamá National Park on 27 June 1999 (IAvH-A 10664) and 3 September 2008 (IAvH-A 14948). In the municipality of Piedecuesta, Santander, a fledgling was collected at Hacienda Las Vegas on 23 September 1949 (USNM 411791) and another was seen at at Finca Ramírez, vereda Parra Juan Rodríguez, on 13 July 2007 (J.E.A. pers. obs.). A similar periodicity for breeding has been recorded in *Scytalopus griseicollis gilesi* (fledgling on 24 June 2008, ICN 35610) and *Scytalopus griseicollis griseicollis* (nestlings and fledglings from June to December, ICN 35441, 36997, 38528, 38529, 373416, IAvH 10305, 12701, 13935, USNM 373416). Breeding periods in *Scytalopus griseicollis* throughout its range could be triggered by the timing of rainy seasons, which present two peaks in the Eastern Cordillera (April-May and September-November) (Morales et al. 2007).

#### Conservation.

*Scytalopus griseicollis* is a range-restricted species mainly associated with patchy cloud forest and páramo of the northern Eastern Cordillera. A scenario of deforestation and habitat fragmentation affects the subpáramo-páramo belt of the entire Colombian Andes (Van der Hammen 2002). The west slope of the Eastern Cordillera, where most of the potentially suitable habitat for *Scytalopus griseicollis morenoi* is found, represents the second most deforested cloud forest region in the Colombian Andes, with only small and more isolated fragments remaining (Morales-R and Armenteras-Pascual 2013). An unexpected small scale forest recovery was observed in the northern Andes (Eastern and Central Cordilleras) between 2001 and 2010, although this may have been influenced by the then prevailing security situation. Any increase in northern Andean Páramo has been slight and may have been wholly offset by expansion of potato plantations in the departments of Boyacá and Santander (Sánchez-Cuervo et al. 2012). Moreover, projected climate change is modelled to be particularly acute for higher elevations of the northern Eastern Cordillera in future (Velásquez-Tibata et al. 2012). The new subspecies occurs in few protected areas that have a broad elevational range (which might mitigate such threats). *Scytalopus griseicollis morenoi*, and the species as a whole, have doubtless suffered a significant reduction of potential area of occupancy as a result of man’s influence on the habitats of the Eastern Cordillera. The largest national park established to protect East Andean montane forests, in Serranía de los Yariguíes, does not protect the new subspecies – which is replaced there by *Scytalopus griseicollis gilesi*. Opportunities to expand other protected areas in the main East Andean range and to promote habitat connectivity in the region would be welcome (Sánchez-Cuervo et al. 2012). Several protected areas in the northern Eastern Cordillera, such as the Tamá binational National Park and the Páramo de Santurbán and Bosques Andinos Húmedos El Rasgón Regional Parks could harbour important populations of this subspecies. However, even these protected areas are threatened by a lack of on-the-ground protection measures or park staff and mining proposals.

This subspecies is locally abundant in well conserved high-Andean forests and páramos (Stiles and Rosselli 1998, Donegan and Avendaño 2008), and even in small and fragmented patches of habitat (Echeverry-Galvis and Morales-Rozo 2007, Peraza 2011), which suggests that local populations could resist extinction if some vegetation cover and connectivity is maintained. Further studies of the forests and organisms of the northern Eastern Cordillera are needed to clarify the potential ecological/geographic barriers which isolate different distinctive subspecies of the region (Avendaño et al. 2013) and to establish conservation priorities.

## Discussion

Only eight of out 41 known species of *Scytalopus* exhibit geographic variation in plumage that has been recognised taxonomically at the subspecies level (Krabbe and Schulenberg 1997, 2003, Remsen et al. 2015). This suggests that plumage geographic variation within *Scytalopus* species is more the exception than the rule. However, several morphologically diagnosable populations occur in the Andes of Colombia which are either not fully diagnosable by vocal characters or which vary only in few vocal characters and respond to playback of one another (Donegan and Avendaño 2008, Donegan et al. 2013, López-O et al. 2013). This indicates that intraspecific geographical variation in the genus may have previously been overlooked and should be studied further in other species.

In Donegan and Avendaño (2008), we deferred describing *Scytalopus griseicollis morenoi* due to the need for a more complete understanding of plumage variation and distribution of this population, particularly given the possibility of post-mortem colour changes, commonly referred to as ‘foxing’, which is prevalent in some *Scytalopus* (Krabbe and Schulenberg 1997). The type series of *Scytalopus griseicollis morenoi* is sufficiently fresh to rule out foxing as the cause of the observed differences in dorsal plumage.

Geographical plumage variation in *Scytalopus griseicollis* is mainly in the hue of gray and brown in the underparts and upperparts, which are features that are considered influenced by differences in melanin concentration (Meunier et al. 2010, Galván and Møller 2013). The evolution of these plumage differences could be related to differences in humidity between populations’ distributions, according to Gloger’s rule (Donegan and Avendaño 2008). Certainly, the coincidence of darker populations (here, subspecies *Scytalopus griseicollis gilesi*) in the very humid Yariguíes mountains reflects a pattern observed in several other species, including *Anisognathus lacrymosus* (Donegan and Avendaño 2010). Further work is necessary to determine if geographic variation in *Scytalopus griseicollis* and other members of the genus is correlated with selection pressures related with Gloger’s rule such as thermoregulation (Walsberg 1983), background matching (Zink and Remsen 1986) or resistance to bacterial degradation (Burtt and Ichida 2004).

## Supplementary Material

XML Treatment for Scytalopus griseicollis morenoi

## Supplementary materials

Supplementary material 1 Specimens of *Scytalopus griseicollis* Data type: species data Explanation note: Specimens of *Scytalopus griseicollis* examined at the American Museum of Natural History (AMNH), Colección Ornitológica Phelps (COP), Instituto Alexander von Humboldt (IAvH-A), Instituto de Ciencias Naturales (ICN), Museo de Historia Natural LaSalle (MLS), Museum National d’Histoire Nacional (MNHN); the National Museum of Natural History, Smithsonian Institution (USNM), and the Natural History Museum, Tring (BMNH). This dataset is made available under the Open Database License (http://opendatacommons.org/licenses/odbl/1.0/). The Open Database License (ODbL) is a license agreement intended to allow users to freely share, modify, and use this Dataset while maintaining this same freedom for others, provided that the original source and author(s) are credited.Jorge Enrique Avendaño, Thomas M. Donegan

Supplementary material 2 List of localities of *Scytalopus griseicollis* Data type: occurence data Explanation note: List of localities of *Scytalopus griseicollis* (geographical coordinates in decimal degrees and elevation in meters above sea level) used in ecological niche modelling analysis (Fig. 6). Acronyms for sound archives (CSA, Colección de Sonidos Animales-Instituto Alexander von Humboldt; XC, xeno-canto) and museums (IAVH-A, Colección de aves-Instituto Alexander von Humboldt; ICN, Instituto de Ciencias Naturales of Universidad Nacional de Colombia; COP, Colección Ornitológica Phelps). This dataset is made available under the Open Database License (http://opendatacommons.org/licenses/odbl/1.0/). The Open Database License (ODbL) is a license agreement intended to allow users to freely share, modify, and use this Dataset while maintaining this same freedom for others, provided that the original source and author(s) are credited.Jorge Enrique Avendaño, Thomas M. Donegan
